# Brain N-Glycosylation and Lipidomic Profile Changes Induced by a High-Fat Diet in Dyslipidemic Hamsters

**DOI:** 10.3390/ijms24032883

**Published:** 2023-02-02

**Authors:** Beatrix Paton, Elisabet Foguet-Romero, Manuel Suarez, Jordi Mayneris-Perxachs, Noemí Boqué, Antoni Caimari, Núria Canela, Pol Herrero

**Affiliations:** 1 Eurecat, Centre Tecnològic de Catalunya, Centre for Omic Sciences, Joint Unit Eurecat-Universitat Rovira i Virgili, Unique Scientific and Technical Infrastructure (ICTS), 43204 Reus, Spain; 2 Nutrigenomics Research Group, Departament de Bioquímica i Biotecnologia, Universitat Rovira i Virgili, 43007 Tarragona, Spain; 3 Department of Diabetes, Endocrinology and Nutrition, Girona Biomedical Research Centre (IDIBGI), Hospital Universitari de Girona Doctor Josep Trueta, 17190 Girona, Spain; 4 CIBER Pathophysiology of Obesity and Nutrition (CIBEROBN), Instituto de Salud Carlos III, 28029 Madrid, Spain; 5 Eurecat, Centre Tecnològic de Catalunya, Technological Unit of Nutrition and Health, 43204 Reus, Spain; 6 Eurecat, Centre Tecnològic de Catalunya, Biotechnology Area, 43204 Reus, Spain

**Keywords:** dyslipidemia, mediterranean diet, high-fat diet, lipidomics, brain glycosylation, N-glycan, mass spectrometry

## Abstract

The consumption of diets rich in saturated fats is known to be associated with higher mortality. The adoption of healthy habits, for instance adhering to a Mediterranean diet, has proved to exert a preventive effect towards cardiovascular diseases and dyslipidemia. Little is known about how a suboptimal diet can affect brain function, structure, and the mechanisms involved. The aims of this study were to examine how a high-fat diet can alter the brain N-glycan and lipid profile in male Golden Syrian hamsters and to evaluate the potential of a Mediterranean-like diet to reverse this situation. During twelve weeks, hamsters were fed a normal fat diet (CTRL group), a high-fat diet (HFD group), and a high-fat diet followed by a Mediterranean-like diet (MED group). Out of seventy-two identified N-glycans, fourteen were significant (*p* < 0.05) between HFD and CTRL groups, nine between MED and CTRL groups, and one between MED and HFD groups. Moreover, forty-nine lipids were altered between HFD and CTRL groups, seven between MED and CTRL groups, and five between MED and HFD groups. Our results suggest that brain N-glycan composition in high-fat diet-fed hamsters can produce events comparable to those found in some neurodegenerative diseases, and may promote brain ageing.

## 1. Introduction

A large portion of the world population has adopted unhealthy eating habits that can undermine healthcare systems unless current trends are inverted towards more sustainable lifestyle models [1]. In 2017, 11 million deaths were attributed to dietary risk factors, confirming that a suboptimal diet has an evident impact on mortality across nations [2]. 

A direct relationship exists between dietary patterns and obesity/overweight risk [3]. Therefore, it is important to understand the mechanisms that link diet with obesity, to find new effective preventable measures. Research has shown that obesity disturbs brain structure and function [4], and is associated with brain-level molecular changes [5]. Dietary interactions with the hypothalamus appear to be key in the development of obesity. Exposure to a high-fat diet (HFD) influences the proteome of the hypothalamus, showing cellular stress, altered synaptic plasticity, and altered mitochondrial function [6]. Moreover, the contribution of insulin resistance, essential fatty acid consumption, and oxidative stress, coordinated with inflammatory and vascular alterations, cause overall changes in brain function with the consumption of high-fat and high-glycemic index-type diets [7]. 

Recent evidence supports the idea that the microbiome could be poorly affected by HFDs, resulting in a state of dysbiosis, leading to increased insulin resistance and inflammation [8]. Dysbiosis is not only associated with gastrointestinal disorders but also with diseases affecting other distal organs. For instance, the nervous system and the gastrointestinal tract communicate through a bidirectional network of signaling pathways known as the gut-brain axis. However, numerous mechanisms behind the impact of the gut microbiota in neuro-development and -pathogenesis remain poorly understood [9].

An effective approach to improving lifestyle and decreasing risk factors contributing to non-communicable diseases can be obtained with adherence to a dietary plan inspired by the principles of the Mediterranean Diet (MED) [1,10]. The MED is characterised by the high consumption of foods and nutrients presumed to be healthy for the organism and specifically for the brain, such as vegetables, fruits, fish, unsaturated fatty acids, and diverse antioxidants [11].

Adherence to the MED has been associated with a lower risk of obesity [12], cardiovascular mortality [13], and type-2 diabetes [14], amongst other diseases. Furthermore, compliance with MEDs has also been associated with slower cognitive decline [15] and a reduction in the incidence of neurodegenerative disorders such as Parkinson’s disease [16] and Alzheimer’s disease [17]. For instance, the increasing consumption of antioxidant-rich foods has been associated with better cognitive performance in elderly subjects at high cardiovascular risk [18]. 

Glycans are essential functional components that participate in numerous physiological processes aimed to maintain organ function and homeostasis in living organisms [19]. N-glycosylation has a wide variety of essential modulatory roles in glycoproteins that are involved in nervous system development and functioning [20]. This post-translational modification influences neuronal excitability and behaviour by affecting voltage-gated ion channels [21] and also alters neurotransmitter receptors, such as the α-amino-3-hydroxy-5-methyl-4-isoxazolepropionic acid receptor (AMPAR) [22] and the N-methyl-D-aspartate receptor (NMEDAR) [23]. More specifically, the loss of core fucosylation on AMPARs enhances their heteromerization, increasing sensitivity for postsynaptic depolarization and consequently activating NMEDARs. As a result, this event impairs long-term potentiation, which is closely related to learning and memory in the hippocampus [24]. 

Furthermore, N-glycosylation in the hippocampus is required for the consolidation and reconsolidation of contextual fear memory in mice [25]. However, research on how a HFD affects N-glycosylation is limited. Barboza et al. suggested that chronic consumption of HFD and induced obesity results in abnormal hippocampal cell surface N-glycosylation [26].

Lipids also have essential roles in the nervous system. They are essential for cellular functioning due to their role in membrane composition, signalling, and energy metabolism. The brain is the second most abundant organ in terms of lipid concentration and diversity, only after adipose tissue [27]. The most representative lipid subtypes in the central nervous system are glycerophospholipids, sphingolipids, and sterol lipids. Altered lipid metabolism has been associated with structural damage, inflammatory processes, apoptotic signalling, and increased oxidative stress resulting in neural impairment leading to neurodegeneration [28]. Furthermore, a higher prevalence of obesity is known to increase the risk of developing several neurological disorders which have been associated with the increase of certain lipid species. For instance, in Alzheimer’s disease, HIV, arteriosclerosis, and ageing, long-chain ceramides are found to be increased [29].

An unparalleled level of complexity exists in the development, organisation, and regulation of the brain, in which N-glycosylation plays a critical role, highlighting the need to identify and understand the unique N-glycan species involved. In line with our interest in obtaining a complete picture of brain protein N-glycosylation, the main aim of this study is to investigate how the brain N-glycan and lipid profile can be affected by a HFD, and evaluate the changes induced when switching to a Mediterranean-like diet after consuming a HFD. This research could shed more light on the plasticity of the brain and its capacity to recover when individuals are subjected to opposing and sequential lifestyle routines using a HFD followed by a switch to a healthy regime based on a Mediterranean-like diet. Golden Syrian hamster was selected for its similar response to humans in terms of lipoprotein metabolism and aortic lesion morphology when fed high cholesterol and saturated fat diets [30]. 

## 2. Results and Discussion

In the present study, we hypothesised that the intake of a Mediterranean-like diet after the consumption of a HFD would ameliorate or reverse the alterations in the brain N-glycan and lipid profile, produced by the HFD, similar to what has been observed in liver steatosis [31]. The experimental design employed attempts to resemble, to some extent, a situation in which subjects showing a high adherence to unhealthy diets are advised to shift towards a healthier diet, such as the MED, to ameliorate alterations or diseases strongly associated with unbalanced diets.

### 2.1. Body Composition, Serum Analyses, and Histological Evaluation

**Figure 1:**
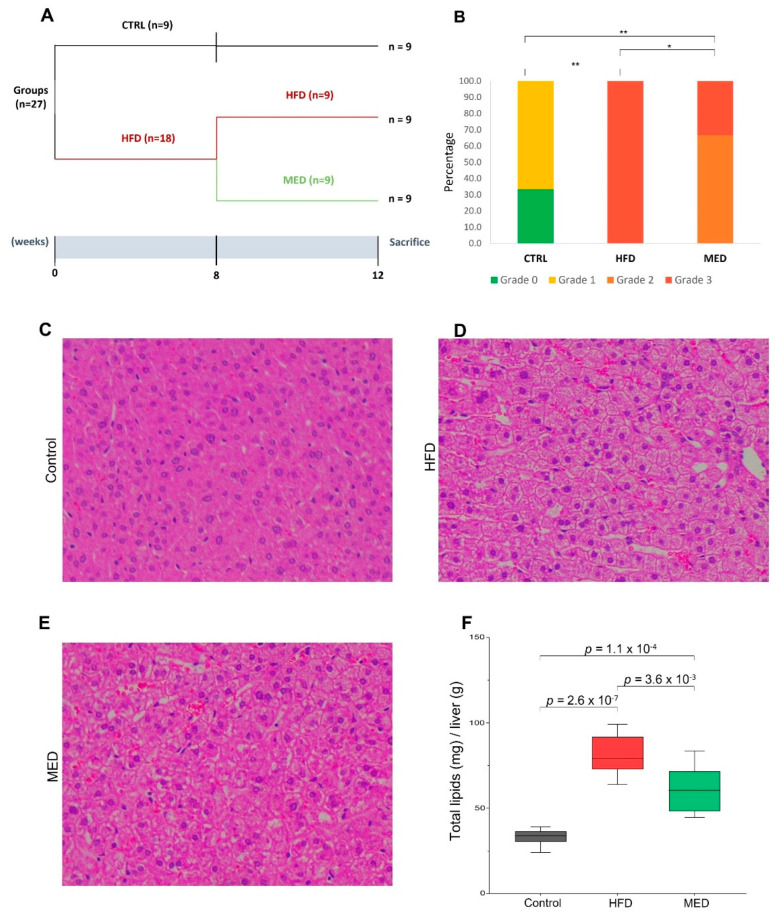
Effect of the HFD and MED on liver histology of Golden Syrian hamsters. (**A**) Outline of the experimental design. (**B**) Steatosis score of histological changes in the liver. Differences were detected using Fisher’s exact test (* *p*-value < 0.05, ** *p*-value < 0.001). (**C**–**E**) Histological analysis of steatosis in liver sections stained with Hematoxylin and Eosin (400×). (**F**) Relative hepatic lipid content of hamsters in experimental groups. CTRL, normal diet; HFD, high-fat diet; MED, Mediterranean-like diet. Differences among groups in hepatic total lipid content were detected by Student *t*-test. All results were considered statistically significant at *p*-value < 0.05.

We assessed the changes in body weight, and biometric and serum variables of each hamster group at week 8 and week 12, as well as examining the phenotypic differences in all groups. Week-8 hamsters received either a NFD or HFD, whereas week-12 hamsters received either a complete HFD (HFD group), a complete NFD (CTRL group), or an 8-week HFD followed by a 4-week MED (MED group) (Figure 1A). As predicted, total cholesterol levels significantly increased in the HFD group compared to the CTRL group after 8 weeks (Table 1). No significant changes were found at this time between groups either in cumulative energy intake or in the biometric parameters (Table 1). At the endpoint (week 12), HFD hamsters displayed increased circulating levels of total cholesterol, low-density lipoprotein (LDL), and high-density lipoprotein (HDL) compared to the CTRL group (Table 2). The shift to a Mediterranean-like diet did not dampen the increase observed in the serum levels of lipids observed after the HFD challenge (Table 2). No differences were observed in final body weight (*p* = 0.586) in HFD-fed and CTRL animals, which can be partially attributed to a trend towards lower caloric intake in HFD-fed animals (Table 2). However, the HFD group showed significantly increased mesenteric white adipose tissue depot (MWAT) weight compared to the CTRL group, an increase that was also observed in the MED hamsters versus their CTRL counterparts. A higher caloric intake was observed in the MED hamsters compared to the HFD group (Table 2). 

In comparison with the CTRL group, the HFD hamsters also developed microvesicular steatosis without apparent inflammation or fibrosis (Figure 1C,D). In agreement with the histological analyses, the HFD group displayed a significant increase in lipid content compared to their control counterparts (Figure 1C). We observed that, at week 12, hamsters fed with the HFD had a significantly more severe grade of hepatic steatosis (measured as steatosis score after histological analysis) compared to those from the CTRL group (*p* < 0.001), and this HFD-induced hepatic steatosis was significantly attenuated in MED hamsters in comparison to their HFD counterparts (Fisher’s exact test; *p*-value < 0.05) (Figure 1B). The shift to a Mediterranean-like diet for 4 weeks significantly decreased fatty liver microvesicular steatosis and, accordingly, decreased the total hepatic lipid content in the MED animals in comparison with the HFD hamsters (Figure 1F).

### 2.2. N-Glycosylation

**Figure 2:**
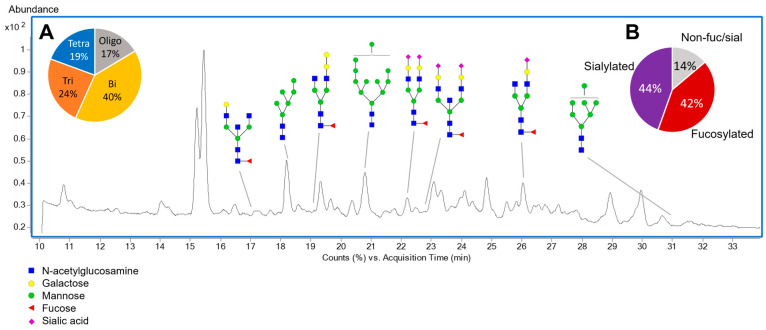
Representative chromatogram of the brain N-glycan profile representing selected statistically significant structures in the three groups (CTRL, HFD, and MED). (**A**) Pie chart depicting the degree of the 72 N-glycans identified in hamster brain tissue. Oligo, oligomannose; Bi, biantennary; Tri, triantennary; Tetra, tetrantennary. (**B**) Pie chart depicting the percentage of fucosylated and sialylated N-glycans in hamster brain tissue. Non-fuc/sial, non-fucosylated and non-sialylated N-glycans. Structural symbols for the N-glycans are shown below the chromatogram.

Total brain N-glycan composition was determined and compared between groups. The Oxford nomenclature was used to annotate individual glycan structures where A represents the number of antennae present, F indicates the fucose, B indicates the presence of a bisecting N-acetylglucosamine, G represents galactoses, and S denotes sialic acids [32]. Figure 2 shows the N-glycan profile in brain tissue. Each identified glycan with its corresponding monoisotopic mass and composition can be found in Table S5.

A high correlation exists between the glycosylation profiles in human and mouse brains, indicating little interspecies variation in brain glycosylation [33]. Complex/hybrid-fucosylated glycans have been reported to be the most abundant glycan class in the brain. Higher levels of fucosylation and lower levels of sialylation have been observed in the brain compared with serum [33,34]. The high content of high-mannose glycans is also characteristic of the brain glycosylation pattern. Oligomannosides tend to be eliminated during the processing of sugars to yield mature N-glycans, except in the brain, where they are carried to the cell surface on recognition molecules [35]. 

Several glycan classes, including mannose, complex, and tentative hybrid types were identified in our brain hamster samples. Two levels of confidence were used to assign the certainty with which a feature was assigned an N-glycan. The lowest level of confidence comprised 32 compounds, identified through Simglycan software. The composition of 2 additional compounds was determined using Simglycan and Glycostore. Lastly, with the highest degree of confidence, the composition and structure were identified for 38 N-glycans employing Simglycan and Glycostore. It is important to note that the use of the RFMS kit provided high intensities and high signal enhancement for neutral glycans, consequently allowing the identification of a large amount of N-glycans. 

Regarding the research on the influence of a HFD and a MED, we found some statistically significant differences in N-glycans between HFD and MED vs. CTRL, but no relevant differences between MED and HFD. These results suggested that the HFD, which was administrated before the MED nutritional intervention, exerted a high impact on glycan composition compared to the second nutritional intervention with a MED. In more detail, the statistical analysis revealed statistically significant changes in fourteen N-glycans (*p*-value < 0.05) for the HFD group vs. CTRL group, nine for the MED group vs. CTRL group and, only one for the MED group vs. HFD group. The composition and structure of the statistically significant N-glycans are shown in Table 3. Complex N-glycans accounted for approximately 83% of these statistically significant features, while high mannose glycans accounted for approximately 17%.

**Figure 3:**
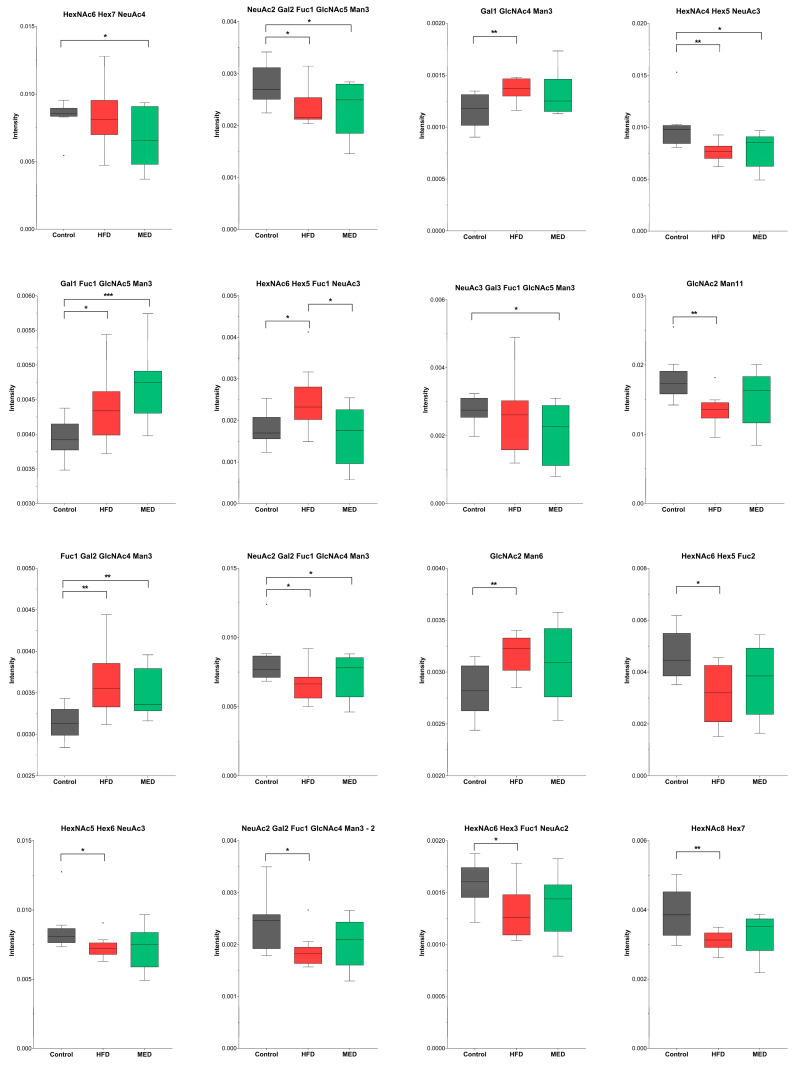

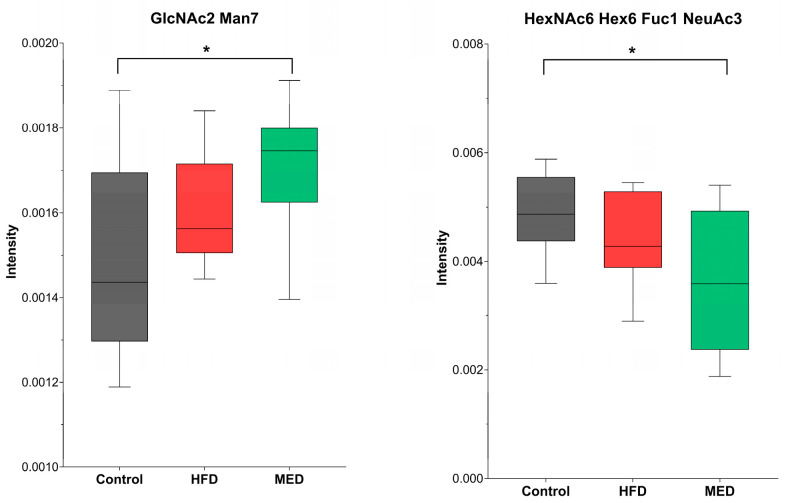
Boxplots showing increased and decreased abundance at week 12 for each statistically significant N-glycan (* *p*-value < 0.05, ** *p*-value < 0.01, *** *p*-value < 0.001).

Remarkably, all the statistically significant sialylated glycans were increased in the CTRL group (vs. HFD and MED group), except for N-glycan HexNAc6Hex5Fuc1NeuAc3 (Table 3). Sialylated structures such as F(6)A2BG(4)2S(6,6)2 and F(6)A2G(4)2S(6,6)2 were increased in the CTRL, whereas non-sialylated structures such as F(6)A2BG(4)1 and F(6)A2G2 were decreased in the CTRL group compared to HFD and MED groups, indicating that the Mediterranean-like diet was not able to reverse the effect of the HFD. In Figure 3, boxplots of all the statistically significant N-glycans for CTRL, HFD, and MED groups are shown. A similar tendency was observed in a study where HFD-fed mice showed a significant decrease in complex tri- and tetraantennary sialofucosylated N-glycans in the hippocampus brain region. Interestingly, transcript analysis of the glycosyltransferases showed that the *MGAT2* gene, which encodes the enzyme responsible for initiating the synthesis of complex branched N-glycans, was significantly decreased in hippocampi of HFD-fed obese mice compared to lean controls [26].

Moreover, statistically significant differences were observed in three oligomannose glycans. They were increased in both the HFD group and MED group compared to the CTRL group, except for M11 which was decreased in the HFD group vs. the CTRL group. Regarding fucosylation, nine structures were increased in the CTRL group compared to the HFD and MED groups, whereas five fucosylated structures showed the opposite tendency, increasing in the HFD and MED groups compared to the CTRL group (Figure 3). Only one glycan (HexNAc6Hex5Fuc1NeuAc3) was found to be statistically significant between the MED and HFD groups. This sialylated and fucosylated glycan increased in the HFD group compared to the CTRL and MED groups, showing an opposite tendency compared to the rest of statistically significant N-glycans (Figure 3). 

Overall, these results show that the four-week nutritional intervention with a MED in our study was not able to have an effect on brain N-glycan composition compared to hamsters fed with a HFD. However, the study demonstrates that the HFD influences brain N-glycan composition. Thus, most sialylated and fucosylated structures are decreased in the HFD-fed group, whereas most oligomannose N-glycans are increased. These tendencies have been observed in other studies where N-glycan galactosylation, sialylation, and fucosylation events decrease with ageing. Abnormal N-glycan alterations have previously been associated with chronic consumption of HFD and induced obesity, specifically in the hippocampal brain region [26]. Similar alterations have been determined in age-related diseases, such as Alzheimer’s disease (AD), amyotrophic lateral sclerosis (ALS), or Parkinson’s Disease (PD). For instance, increased levels of sialylated glycans have been found in ALS [36] but decreased in AD [37] and PD patients [38]. Similar to our results, fucosylation decreases in T2DM [39] and ALS patients [36], but increases in PD [38]. Taking this into account, our results suggest that brain N-glycan behaviour related to adherence to a HFD can produce events similar to those found in some neurodegenerative diseases and may promote brain ageing process.

### 2.3. Lipidomics 

In the evaluation of the brain lipid profile, 192 lipids were identified and quantified (as relative concentrations) in the different groups. The identified lipids belong to the following species: Lysophosphatidylcholines (LPC), lysophosphatidylethanolamines (LPE), phosphatidylcholines (PC), phosphatidylethanolamines (PE), sterol esters (SE), sphingomyelins (SM), triglycerides (TAG), and diacylglycerides (DAG). Each brain region has differences in the lipid pattern. Specifically, the hippocampus and the cortex have a similar pattern, however, the olfactory bulb and the hypothalamus show different lipid profiles [40]. The hypothalamus is characterised as being enriched in fatty acids as it has a high cell density, and therefore a high content of structural lipids such as phospholipids and cholesterol esters [41]. This is reflected in our data as LPC, LPE, PC, PE, and SM were the most abundant lipids found in our hamster brain samples. 

Statistically significant differences (*p* < 0.05) were observed in forty-nine lipids in HFD samples vs. CTRL samples, whereas only seven lipids showed a significant change in MED samples vs. CTRL and, five lipids in MED samples vs. HFD samples (Table 4). These results suggest that a HFD clearly alters the brain lipid profile and that the 4-week MED nutritional intervention after a HFD barely modifies the lipid profile. In the present study, PC werethe most abundant lipids in the brain, in concordance with previous results [40]. Two PC (34:2 and 36:5) were increased in the HFD and MED groups compared to the CTRL group. Moreover, two PE (30:1 and 38:2) were increased in the HFD group compared to the CTRL and the MED group. Both PC and PE are the most abundant phospholipids in all mammalian cell membranes. Abnormally high and low cellular PC/PE ratios are known to influence energy metabolism and can indicate disease progression [42]. 

Two types of glycerolipids, specifically TAG and DAG, were altered in the different groups. TAG 50:1, 52:1, and 56:5 were increased in the HFD group compared to the CTRL group, and, DAG 36:4 and 38:4 were increased in the HFD group compared to the MED group. A similar trend has been observed in other studies which demonstrated that high-fat feeding increases the total contents of TAG and DAG in the hypothalamus [29,40]. Moreover, DAG have been reported to be the lipid species most influenced by a HFD, showing a significant increase in four different brain regions [40]. Taking this into account, our results suggest that the MED may have a positive impact on the brain DAG levels in animals previously subjected to a HFD. However, previous studies have shown that the lipid profile of the hypothalamus isnot affected by a HFD, suggesting that the hypothalamus cannot be a depot for dietary lipids, as the blood barrier serves as a metabolic shield to the brain [41]. This could explain why, in our study, DAG levels were not altered in the HFD group vs. the CTRL group. These non-consistent results between different groups could be a result of having characterised the whole brain tissue instead of focusing on profiling one specific brain region. 

Lysophospholipids were increased in the HFD group compared to the CTRL group and decreased in the MED group compared to the CTRL group. This obtained data supports a different study which determined that a HFD affected lysophospholipids in the cortex and hippocampus, but did not affect the hypothalamus and olfactory bulb [40]. Moreover, circulating levels of lysophospholipids have been reported to change with the progression of dyslipidemia-related diseases [43]. In our study, brain LPC and LPE increased in the HFD group compared to the CTRL group. Similar shifts in plasma and hepatic levels of LPC and LPE have been associated with chronic intake of a HFD in obese [44] and non-obese mice [45]. More specifically, in our study, LPE 20:4, a lipid reported to exhibit early alterations in response to dyslipidemia, was decreased in the MED compared to the CTRL group. Consistent with previous reports, LPC and LPE, specifically LPE 20:4, could serve as potential biomarkers of risk of developing lipid disorders [43].

## 3. Materials and Methods

### 3.1. Reagents 

Trichloroacetic acid (TCA), acetone, ammonium bicarbonate (ABC), ammonium formate, chloroform, sodium chloride (NaCl), and formic acid (LC-MS grade) were purchased from Sigma-Aldrich (St. Louis, MO, USA). RIPA Lysis and Extraction Buffer were obtained from ThermoFisher Scientific (Waltham, MA, USA), and, 2-propanol (LC-MS grade), methanol (LC-MS grade), acetonitrile (LC-MS grade), and methyl-*tert*-butyl ether were purchased from Merck (Darmstadt, Germany). The water used throughout the study was purified with a Milli-Q system from Millipore (Burlington, MA, USA).

### 3.2. Diets

A normal-fat diet (NFD) and two isocaloric diets, a HFD and a ME-like diet were used (ENVIGO, Barcelona, Spain) (Table S1). The NFD contains 11% calories from fat, whereas the HFD contains 23% calories from fat and 1 g/kg cholesterol. In the MED (23% calories from fat), extra virgin olive oil (EVOO; 50 g/kg of diet), walnuts (20 g/kg of diet), and fish oil (6 g/kg of diet) were added to the HFD in place of lard. In addition, in the MED, total cholesterol content was adjusted to 250 mg/kg of diet, taking into account the amount of cholesterol present in lard and fish oil. The nutritional composition and manufacturing companies of the ingredients used in the MED are specified in Tables S2–S4. 

Considering an average hamster’s weight of 130 g and a daily diet intake of 7 g, the amounts of EVOO, walnuts, and fish oil included in the MED were equivalent to the daily consumption of 26.8 g, 10.7 g, and 0.97 g of these food items, respectively, for a 60 kg human. These daily amounts can be usually achieved in the context of a Mediterranean diet pattern [46]. In the MED, cholesterol was included at 0.025%, because, according to previous research, it is non-atherogenic for hamsters at this concentration [47,48]. The extrapolated daily intake using the same formula was 134 mg, which is lower than the daily cholesterol intake estimated in the PREDIMED study (340 mg/day) [46]. 

### 3.3. Animals and Experimental Design 

The Animal Ethics Committee of the Technological Unit of Nutrition and Health of Eurecat (Reus, Spain) and the Generalitat de Catalunya approved all procedures (DAAM 10026). The experimental protocol complied with the ARRIVE guidelines followed the ‘Principles of laboratory animal care’ and was carried out in accordance with the EU Directive 2010/63/EU for animal experiments. All animals were housed individually at 22 °C under a light/dark cycle of 12 h (lights on at 09:00 a.m.) and were given free access to food and water. Twenty-seven 10-week-old male Golden Syrian hamsters (Janvier Labs, Saint Berthevin, France) weighing 110–120 g were used. After an adaptation period of 1 week, hamsters were randomly assigned into two experimental groups fed with a normal fat diet (CTRL group, fed the NFD, n = 9, 11% calories from fat; Envigo, Barcelona, Spain) or a HFD (n = 18, 23% calories from fat and 1g/kg cholesterol; Envigo, Barcelona, Spain) for 8 weeks. In a previous study, using similar diets, we demonstrated that this period was useful to induce fatty liver and hypercholesterolemia in hamsters [31,49]. Afterwards, the HFD group was randomly distributed into two subgroups. The first subgroup continued receiving a HFD until week 12 (HFD group, n = 9). The second subgroup changed to the MED during the last 4 weeks of the study (MED group, n = 9) (Figure 1A). The sample size was calculated with the statistical program G*Power (version 3.1.9.4). Based on the LDL levels and their standard deviations, with a statistical power of 90%, a confidence level of 95%, and carrying out a Student *t*-test, the minimum sample size necessary to detect a difference of at least 10% in LDL cholesterol levels was eight animals per experimental group. To cover possible interferences in the response within each group, nine animals per group were finally selected.

Body weight and food intake were recorded once per week, and food was renewed daily. At 12 weeks, all experimental animals were sacrificed under anaesthesia (pentobarbital sodium, 60 mg/kg body weight) after 6 h of diurnal fasting. Blood was collected by cardiac puncture, and serum was obtained by centrifugation and stored at −20 °C until analysis. The liver, MWAT, and brain were rapidly removed, weighed, frozen in liquid nitrogen, and stored at −70 °C until analysis.

### 3.4. Histological Evaluation

Morphometric analyses of tissues and steatosis of liver histology were carried out using the methods described in the literature [31]. Briefly, liver samples (n = 9 per group) were fixed, and subjected to dehydration and paraffin infiltration-immersion. After obtaining the tissue sections, a histological examination was carried out. The histological sections of the liver were analysed according to the Kleiner scoring system [50]. 

### 3.5. Hepatic Lipid Extraction and Quantification

Lipid extraction and quantification were carried out using the methods previously described [31]. Briefly, liver samples (80–100 mg) were mixed with 1 mL of hexane:isopropanol (3:2, *v*/*v*). The tubes with the samples were gassed with nitrogen before being closed to minimize lipid oxidation and then left overnight under orbital agitation at room temperature protected from light. The content of each tube was transferred into a new one, and 0.3 mL of Na_2_SO_4_ (0.47 M) was added. Tubes were mixed for 5 min, left for 15 min in orbital agitation, and centrifuged at 1000× *g* for 10 min at 4 °C. The upper phase containing lipids was dissolved in hexane and transferred to a clean, previously weighed glass tube. The hexane extract was then dried with nitrogen gas. Once the tube was dried, the percentage of lipids was determined gravimetrically.

### 3.6. Body Composition Analyses

Body composition was analysed by Nuclear magnetic resonance (NMR). Lean and fat mass analyses were performed at the end of weeks 8 and 12 using an EchoMRI-700^®^ device (Echo Medical Systems, L.L.C., Houston, TX, USA). The measurements were performed in duplicate. Data are expressed in relative values as a percentage of body weight (%). The lean/fat mass ratio was also calculated.

### 3.7. Serum Analysis

Enzymatic colorimetric assays were used for the analysis of glucose, total cholesterol and triglycerides (QCA, Barcelona, Spain), HDL-cholesterol, and LDL/VLDL-cholesterol (Bioassay systems, Hayward, CA, USA). Serum insulin levels were analysed using a hamster insulin ELISA kit (MyBiosource, Bizkaia, Spain).

### 3.8. N-Glycan Analysis 

#### 3.8.1. Protein Extraction and Quantification

Samples containing approximately 65 mg of brain tissue were resuspended in 1 mL of RIPA buffer and subjected to bead beating combined with a freeze–thaw cycle. Three to four 1.4 mm stainless steel beads were added to each sample, followed by a 1 min bead beating cycle at medium speed (Bullet Blender, Cultek, Barcelona, Spain); this step was repeated twice. Samples were then agitated for 1.5 h at 20 °C and centrifuged at 16,000× *g* for 20 min. Proteins in the supernatant were precipitated overnight with TCA/acetone. The obtained pellet was resuspended in 50 mM ABC and the concentration was quantified using the Bradford method.

#### 3.8.2. De-N-Glycosylation and Labelling of N-Glycans

Sample denaturation, de-N-glycosylation, labelling with Rapifluor-MS (RFMS), and purification were performed in accordance with the Waters Corporation “GlycoWorks RapiFluor-MS N-Glycan Kit Care and Use Manual” (p/n 715004793) (Waters, Milford, MA, USA). Briefly, the glycoproteins were denatured at 90 °C for 3 min in the presence of 5% (*w*/*v*) RapiGest. De-N-glycosylation was then conducted at 50 °C for 5 min, with the addition of 1.2 μL of Rapid PNGase F. The digested samples were directly subjected to RFMS labelling without purification by adding 6 μL of RFMS reagent. The reaction was then allowed to proceed at room temperature for 5 min. A GlycoWorks μElution Plate was used for the SPE Clean-up procedure. Glycans were eluted with 200 mM ammonium acetate in 5% acetonitrile.

#### 3.8.3. LC-MS/MS

Derivatized samples were analysed using an Agilent UHPLC 1290 Infinity Series coupled to an Agilent qTOF/MS 6550 Series (Agilent Technologies, Santa Clara, CA, USA). Mobile phase A was 50 mM ammonium formate solution, and mobile phase B was acetonitrile. N-glycans were separated on a Waters ACQUITY UPLC BEH amide column (2.1 mm × 150 mm i.d., particle size 1.7 μm). The flow rate was 0.4 mL/min, the injection volume was 20 µL, and the column temperature was set to 60 °C. Initially, the gradient ramped mobile phase A from 25 to 46%, over 35 min. From 35 to 36.5 min, the gradient ramped from 46 to 100% solvent A and the flow rate was lowered to 0.2 mL/min. A 100% solvent A was held constant from 36.5 to 39.5 min, after which the percentage of solvent A decreased to 25%, from 39.5 min to 43.1 min. The flow rate was then increased back to 0.4 mL/min from 43.1 to 47.6 min and solvent A was held constant at 25%. Lastly, the parameters were held constant from 47.6 to 55.0 min. The qTOF operated in positive electrospray ionisation mode (ESI+), and mass spectra were recorded between m/z 300–1700 at 1.5 spectra/s. The source conditions were 25 psi for nebuliser gas, 200 °C for gas temperature, 12 L/min for gas flow, 250 °C for sheath gas temperature, 12 L/min for sheath gas flow, 3500 V for capillary voltage, and 500 V for nozzle voltage. Additionally, tandem mass experiments (MS/MS) using data dependence acquisition at a collision energy of 30 eV from the 10 most intense ions were used for identification purposes.

#### 3.8.4. Data Processing of LC-MS Data

The MS data were first processed using Agilent MassHunter Qualitative Analysis B.07 software. For each sample, total ion chromatograms (TIC) containing MS/MS fragmentation data were deconvoluted using the “find molecular feature” algorithm to find chromatographic peaks that could potentially be N-glycans. The resulting list of entities containing the MS/MS data was exported and loaded to Simglycan software v.5.93 (Premier Biosoft, San Francisco, CA, USA) for molecular and structural elucidation. Simglycan is a high throughput structural identification tool that uses a built-in database with theoretical fragmentation profiles to provide the most likely structure candidates [51]. The results obtained from Simglycan were matched against the GlycoStore database (https://www.glycostore.org (accessed on 24 March 2022)) to refine the identification results. GlycoStore was able to provide elution property information for over 850 unique structures including standardized retention times, expressed as glucose units (GU), for the RFMS-labelled glycans [52]. After the identification process of N-glycans on brain samples, 72 N-glycans were identified. Then, the exact mass [M + H]^+^, [M + 2H]^2+^ or [M + 3H]^3+^ of these structures were extracted on all samples using Agilent Mass Hunter Quantitative software (B.07) to create a refined matrix of quantitative data for statistical purposes. Finally, the data matrix obtained containing the area of each identified N-glycan was normalized by dividing the peak area of each N-glycan with the sum of the area of all N-glycans in each sample.

### 3.9. Lipidomics Analysis 

#### 3.9.1. Sample Preparation

Samples were extracted using an adapted Folch procedure [53,54,55]. Approximately 150 mg of brain tissue samples were extracted with 1500 µL of chloroform:methanol (2:1, *v*/*v*) containing internal standard mixture (Lipidomix SPLASH^®^, Avanti Polar Lipids, Birmingham, AL, USA) and homogenized using a bead beating method. Next, 300 µL of water with NaCl (0.9%) was added and centrifuged at 15,000 rpm for 10 min to promote liquid phase separation. The lower phase was recovered, evaporated to dryness, and reconstituted with methanol:methyl-tert-butyl ether (9:1) for LC-MS analysis.

#### 3.9.2. LC-MS

Samples were analysed using an Agilent UHPLC 1290 Infinity Series coupled to an Agilent qTOF/MS 6550 Series (Agilent Technologies, Santa Clara, CA, USA). The chromatographic separation consists of an elution with a ternary mobile phase containing water with 10 mM ammonium formate and 0.1% formic acid (solvent A), methanol (solvent B), and 2-propanol (solvent C). The stationary phase was a C18 column (Kinetex EVO C18 Column, 2.6 µm, 2.1 mm × 100 mm) that allows the sequential elution of the more hydrophobic lipids such as lysophospholipids, sphingomyelins, phospholipids, diglycerides, triglycerides, and cholesteryl esters, among others. The flow rate was 0.6 mL/min, the injection volume was 2 µL, and the column temperature was set to 60 °C. The gradient employed was 0–0.5 min, 55–45% A + 10% B; 0.5–1.5 min, 45–42.8% A + 10–9.5% B; 1.5–1.6 min, 42.8–34% A + 9.5–7.5% B; 1.6–5 min, 34–31.8% A + 7.5–7% B; 5–5.1 min, 31.8–18.6% A + 7–4% B; 5.1–7.5 min, 18.6–16.4% A + 4–3.5% B; 7.5–9 min, 16.4% A + 3.5% B; 9–9.5 min, 16.4–0% A + 3.5–0% B; 9.5–11.5 min, 0% A + 0% B; 11.5–11.6 min, 0–45% A + 0–10% B; and 24.75–29.25, 55–45% A + 10% B. The qTOF operated in positive electrospray ionisation mode (ESI+), and mass spectra were recorded between m/z 300–1700 at 3 spectra/s. The source conditions were 35 psi for nebuliser gas, 225 °C for gas temperature, 11 L/min for gas flow, 300 °C for sheath gas temperature, 12 L/min for sheath gas flow, 3500 V for capillary voltage, and 500 V for nozzle voltage.

#### 3.9.3. Data Processing of LC-MS Data

The MS data were processed using both Agilent MassHunter Qualitative and Quantitative Analysis B.07 software. The identification of lipid species was performed by matching their accurate mass and tandem mass spectrum, when available, to Metlin-PCDL from Agilent containing more than 40,000 metabolites and lipids. LipidCreator workbench was also used for targeted exact mass list generation [56]. In addition, chromatographic behaviour of pure standards for each family and bibliographic information was used to ensure their putative identification. After putative identification of lipids, these were quantified in terms of internal standard response ratio using one internal standard for each lipid family by using Agilent Mass Hunter Quantitative software (B.07) to create a refined matrix of quantitative data for statistical purposes.

### 3.10. Statistical Analysis

Differences in energy intake, and biometric and serum parameters between the CTRL hamsters and the HFD hamsters, after 8 weeks, were analysed using Student’s *t*-test. At the endpoint (week 12), Student’s *t*-test was used to evaluate differences in energy intake, hepatic lipid content, biometric parameters, and serum parameters for the three pairs of conditions: HFD vs. CTRL, MED vs. CTRL, and MED vs. HFD. Differences in steatosis scores were evaluated using the Chi-squared test.

The same statistical analysis procedure was employed for both the N-glycan and lipidomics analyses. After the data processing, the normalized data matrices containing 72 identified-glycan-identified entities and 192 lipidic species were separately loaded on Mass Profiler Professional (MPP) software v.15.1 (Agilent Technologies, Santa Clara, MA, USA). Further data normalization was carried out which consisted of transforming data to a log2 scale. To determine the statistically significant differences (*p* < 0.05), a pairwise comparison with Student’s *t*-test was performed between HFD and CTRL, MED and CTRL, and, lastly, MED vs. HFD. 

## 4. Conclusions

In this study, the global brain N-glycome and lipid profile were determined. Using LC-MS/MS, 72 glycans and 192 lipids were identified based on informative MS/MS data. Statistically significant differences were observed in 18 N-glycans and 53 lipids between groups HFD, MED, and CTRL. Results suggested that a short MED diet intervention barely modified the N-glycan and lipid profile on animals that were previously fed with a HFD, confirming that the HFD, administrated before the MED, had more impact on the N-glycan and lipid composition than the MED nutritional intervention itself. The tendencies observed should be confirmed with a longer-term MED after a HFD to elucidate whether the MED can reverse or modify the brain N-glycan and lipid pattern more effectively. 

Regarding the N-glycan profile, most sialylated and fucosylated structures were decreased in HFD-fed groups, whereas most oligomannose structures were increased. These tendencies are commonly observed in ageing studies, suggesting that a HFD can produce events that occur in the ageing process as well as in age-related diseases.

Most of the identified brain lipids belonged to the PC and PE lipid species. PC and PE are the most abundant phospholipids in mammalian cell membranes and more specifically, PC are the most abundant lipids in the brain. Previous studies have reported that certain brain regions are not depots for dietary lipids, as the blood barrier serves as a metabolic shield to the brain. Nevertheless, a statistically significant increase was observed in 48 lipids in the HFD group compared to the CTRL group. Further research could determine if these shifts in the lipid profile entail relevant functional brain changes. Changes observed in both the lipidomic and glycomic brain profile could potentially be caused by the alteration of the gut microbiota after the exposure to a HFD, as a state of dysbiosis would lead to increased insulin resistance and inflammation, leading to an alteration of the nervous system through the gut-brain axis. However, to confirm this hypothesis, studies including metagenomics and metabolomics should be conducted. Additional studies including diets supplemented with antioxidants could also be of interest. Obesity is known to be associated with an increase in cerebral oxidative stress levels, which may enhance neurodegeneration [46]. Some studies have evaluated the neuroprotective action of dietary supplements containing antioxidants (i.e., resveratrol) [47]. In future studies, with HFD-fed animals supplemented with antioxidants, we could assess the modulations induced on lipidomic and glycomic brain profiles to broaden our understanding of the neuroprotection of antioxidants.

## Figures and Tables

**Table 1:** Energy intake, body composition, and serum parameters of hamsters fed with a NFD (CTRL group) or a HFD (HFD group) in the 8th week of the study.

	Control (Week 8) (n = 9)	HFD (Week 8) (n = 18)
**Cumulative food intake (kcal)**	205.45 ± 14.08	198.81 ± 16.78
**Biometric variables**		
Body weight (g)	123.05 ± 5.14	120.76 ± 7.08
Fat mass (%)	11.94 ± 3.20	12.37 ± 2.14
Lean mass (%)	84.89 ± 3.26	84.09 ± 2.12
Lean/fat ratio	7.83 ± 3.22	7.04 ± 1.51
**Serum parameters**		
CHOL (mM) *	4.96 ± 0.66	8.30 ± 0.66

Data are shown as the mean ± SD. * indicates the significant difference (*p* < 0.05) between groups detected by Student’s *t*-test.

**Table 2:** Effects of HFD and MED on food intake and biometric and serum variables at the end of the study (week 12).

	Control (Week 12) (n = 9)	HFD (Week 12) (n = 9)	MED (Week 12) (n = 9)
**Cumulative food intake (kcal) ^a^**	126.8 ± 14.4	118.7 ± 8.3	133.3 ± 9.9
**Biometric variables**			
Body weight (g)	122.20 ± 5.62	119.60 ± 5.71	125.71 ± 10.71
Liver weight (g) ^b,c^	4.23 ± 0.30	5.06 ± 0.43	5.43 ± 0.64
Liver weight (%) ^b,c^	3.49 ± 0.25	4.27 ± 0.30	4.35 ± 0.25
MWAT (%) ^b,c^	0.88 ± 0.24	1.13 ± 0.14	1.23 ± 0.22
MUS	0.27 ± 0.03	0.26 ± 0.03	0.26 ± 0.03
Fat mass (%)	11.28 ± 2.59	11.21 ± 2.12	13.34 ± 2.28
Lean mass (%)	85.42 ± 2.39	85.13 ± 2.17	83.32 ± 2.23
Lean/fat mass ratio	8.00 ± 2.13	7.88 ± 1.71	6.43 ± 1.21
**Serum variables**			
CHOL (mM) ^b,c^	3.33 ± 0.49	6.01 ± 0.51	5.47 ± 0.64
HDL-C (mM) ^b,c^	2.51 ± 0.50	3.78 ± 0.38	3.57 ± 0.73
LDL-C (mM) ^b,c^	0.94 ± 0.27	2.03 ± 0.38	1.97 ± 0.34
TG (mM) ^c^	5.83 ± 1.19	7.10 ± 4.13	10.48 ± 3.09

Data are shown as the mean ± SD. HFD, high-fat diet. MED, Mediterranean-like diet. MWAT, mesenteric white adipose tissue. MUS, gastrocnemius, and soleus muscles. CHOL, total cholesterol. TG, triglyceride. Superscript letters indicate statistically significant differences detected by Student’s *t*-test (*p* < 0.05) between groups; ^a^ Differences for MED vs. HFD, ^b^ differences for HFD vs. CTRL, and ^c^ differences for MED vs. CTRL.

**Table 3:** Statistically significant brain N-glycans by week 12 in the three different pairs of conditions: HFD vs. CTRL, MED vs. CTRL, and MED vs. HFD. HFD, high-fat diet; CTRL, normal-fat diet; MED, Mediterranean-like diet; FC, fold change.

*HFD* vs. *CTRL*					
Glycan Name ^1^	Composition ^2^	Glycan Mass	m/z	*p*	FC
F(6)A2BG(4)2S(6,6)2	NeuAc2 Gal2 Fuc1 GlcNAc5 Man3	2571.92	1442.5620	0.0126	−1.20
A2[6]G(4)1	Gal1 GlcNAc4 Man3	1478.54	895.8720	0.0069	1.19
-	HexNAc4 Hex5 NeuAc3	2513.87	1414.0499	0.0046	−1.28
F(6)A2[3]BG(4)1	Gal1 Fuc1 GlcNAc5 Man3	1827.68	1070.4368	0.0426	1.10
-	HexNAc6 Hex5 Fuc1 NeuAc3	3066.0951	1126.7695	0.0236	1.35
M11 a3D1,[D2(1),D3(1)],a2D4(2)	GlcNAc2 Man11	2206.75	1259.9943	0.0036	−1.32
F(6)A2[6]G1Ga1	Fuc1 Gal2 GlcNAc4 Man3	1786.65	1049.9261	0.0038	1.15
F(6)A2G(4)2S(6,6)2	NeuAc2 Gal2 Fuc1 GlcNAc4 Man3	2368.84	1341.0211	0.0266	−1.23
F(6)A2G(4)2S(3,3)2	NeuAc2 Gal2 Fuc1 GlcNAc4 Man3	2368.84	1341.0211	0.0215	−1.25
M6 D1	GlcNAc2 Man6	1396.49	854.8422	0.0050	1.12
-	HexNAc6 Hex5 Fuc2	2338.8667	1325.9973	0.0103	−1.54
-	HexNAc5 Hex6 NeuAc3	2879.01	1064.7489	0.0366	−1.16
-	HexNAc6 Hex3 Fuc1 NeuAc2	2450.8940	1382.0477	0.0146	−1.23
-	HexNAc8 Hex7	2777.0153	1029.7350	0.0059	−1.24
***MED*** ** vs. *CTRL***					
**Glycan Name**	**Composition**	**Glycan Mass**	**m/z**	***p***	**FC**
F(6)A2BG(4)2S(6,6)2	NeuAc2 Gal2 Fuc1 GlcNAc5 Man3	2571.92	1442.5621	0.0478	−1.22
M7	GlcNAc2 Man7	1558.54	935.8692	0.0310	1.15
-	HexNAc4 Hex5 NeuAc3	2513.87	1414.0499	0.0233	−1.29
F(6)A2[3]BG(4)1	Gal1 Fuc1 GlcNAc5 Man3	1827.68	1070.4368	0.0007	1.19
F(6)A2G(4)2S(6,6)2	NeuAc2 Gal2 Fuc1 GlcNAc4 Man3	2368.84	1341.0211	0.0300	−1.26
-	HexNAc6 Hex6 Fuc1 NeuAc3	3228.15	1180.7875	0.0226	−1.41
F(6)A3G(4)3S(3,3,3)3	NeuAc3 Gal3 Fuc1 GlcNAc5 Man3	1915.69	1113.0953	0.0328	−1.50
-	HexNAc6 Hex7 NeuAc4	3535.24	1283.491	0.0495	−1.30
F(6)A2[6]G1Ga1	Fuc1 Gal2 GlcNAc4 Man3	1786.65	1049.9261	0.0073	1.11
***MED*** ** vs. *HFD***					
**Glycan Name**	**Composition**	**Glycan Mass**	**m/z**	***p***	**FC**
-	HexNAc6 Hex5 Fuc1 NeuAc3	3066.0951	1126.7695	0.0285	−1.59

^1^ Employed glycan nomenclature: F—Fucose; G—Galactose; S—N-Acetylneuraminic acid; Ga—a-linked Galactose; A1—Monoantennary, A2—Biantennary, B, bisecting GlcNAc linked α1-4 to α1-3 mannose. Numbers with parentheses indicate the preceding monosaccharide’s linkage while those not in parentheses indicate the preceding characteristic’s number. For example, F(6)A3G(4)3S(3,3,3)3 represents a core fucosylated triantennary glycan with three galactoses directly attached to antennae, and the three antennae terminated with an N-glycolylneuraminic acid. ^2^ Employed glycan nomenclature for glycan composition: HexNAc—N-Acetylhexosamine; Hex—Hexose; NeuAc—N-Acetylneuraminic acid; Fuc—Fucose; Gal—Galactose; GlcNAc—N-Acetylglucosamine; Man—Mannose.

**Table 4:** Statistically significant differences in brain lipids by week 12 in the three different pairs of conditions. FC, fold change.

*HFD* vs. *CTRL*			
Lipid Species	Compound	*p*	FC
Lysophosphatidylcholines	LPC 14:0	0.0476	1.34
	LPC 18:1	0.0447	1.34
Lysophosphatidylethanolamines	LPE 18:0	0.0307	1.36
	LPE 22:6	0.0239	1.43
Phosphatidylcholines	PC 17:0	0.0498	1.27
	PC 17:1	0.0285	1.30
	PC 30:0	0.0386	1.28
	PC 31:0	0.0255	1.31
	PC 31:1	0.0113	1.37
	PC 32:2	0.0436	1.29
	PC 32:3	0.0259	1.33
	PC 33:0	0.0382	1.29
	PC 34:0	0.0335	1.28
	PC 34:2	0.0057	1.36
	PC 34:5	0.0354	1.35
	PC 35:0	0.0181	1.39
	PC 36:0	0.0239	1.30
	PC 36:2	0.0428	1.26
	PC 36:5	0.0078	1.32
	PC 38:3	0.0408	1.29
	PC 38:5	0.0312	1.41
	PC 39:3	0.0491	1.27
	PC 40:6	0.0295	1.27
	PC 42:2	0.0416	1.85
	PC 42:3	0.0401	1.42
	PC 44:2	0.0167	1.44
Phosphatidylethanolamines	PE 30:1	0.0225	1.47
	PE 36:1	0.0185	1.37
	PE 38:2	0.0355	1.39
	PE 38:5	0.0302	1.33
	PE 38:6	0.0393	1.22
	PE 40:4	0.0458	1.32
	PE 40:6	0.0300	1.26
	PE 42:2	0.0259	1.30
	PE 44:5	0.0265	1.23
Sterol esters	SE 27:1/18:1	0.0196	1.37
	SE 27:1/18:2	0.0013	1.65
	SE 27:1/20:1	0.0008	1.81
	SE 27:1/20:4	0.0027	1.60
	SE 27:1/22:4	0.0339	1.57
	SE 27:1/22:6	0.0401	1.49
Sphingomyelins	SM 32:2;2	0.0434	1.46
	SM 35:1;2	0.0418	1.33
	SM 35:2;2	0.0282	1.31
	SM 40:2;2	0.0283	1.43
Triglycerides	TAG 50:1	0.0497	1.32
	TAG 52:1	0.0091	1.38
	TAG 56:5	0.0163	1.46
***MED*** ** vs. *CTRL***			
**Lipid species**	**Compound**	***p***	**FC**
Lysophosphatidylcholines	LPC 20:4	0.0195	−1.53
Lysophosphatidylethanolamines	LPE 20:4	0.0145	−1.58
Phosphatidylcholines	PC 34:2	0.0375	1.28
	PC 36:5	0.0351	1.27
Sterol esters	SE 27:1/16:1	0.0169	−1.34
	SE 27:1/18:2	0.0399	1.39
	SE 27:1/22:6	0.0010	2.03
***MED*** ** vs. *HFD***			
**Lipid species**	**Compound**	***p***	**FC**
Diacylglycerides	DAG 36:4	0.0494	−1.37
	DAG 38:4	0.0396	−1.40
Phosphatidylethanolamines	PE 30:1	0.0318	−1.36
	PE 38:2	0.0186	−1.38
Sterol esters	SE 27:1/18:3	0.0416	−1.42

## Data Availability

The data presented in this study are available on request from the corresponding author.

## Supplementary Materials

The following supporting information can be downloaded at: https://www.mdpi.com/article/10.3390/ijms24032883/s1.
