# Supplementary material for: Brain N-Glycosylation and Lipidomic Profile Changes Induced by a High-Fat Diet in Dyslipidemic Hamsters
Source: Int J Mol Sci. 2023 Feb 2;24(3):2883. doi: 10.3390/ijms24032883 (PMC9918045; doi:10.3390/ijms24032883)
Supplement: Supplementary file 1 [file ijms-24-02883-s001.zip › ijms-2071745-Supplementary.pdf]

Supplementary Information

Supplementary Table S1. Composition of the diets used in the study. NFD, normal fat diet; HFD, high-fat diet; MED, Mediterranean-like Diet; EVOO, extra-virgin olive oil

	**NFD**	**HFD**	**MED**
**Energy**			
Protein, % of kcal	21.6	21.2	21.3
Carbohydrate, % of kcal	67.2	55.9	55.6
Fat, % of kcal	11.3	22.9	23.1
Kcal/g	3.6	3.9	3.9
**Ingredients (g/kg)**			
Casein	220	233	230
L-Cystine	3	3.2	3.2
Corn Starch	380	291	291
Maltodextrin	100	106	106
Dextrose, monohydrate	50	53	53
Sucrose	100	106	106
Cellulose	50	53	51
Coconut Oil	7.74	5.3	5.3
Flaxseed Oil	5.16	4.2	4.2
Safflower Oil, linoleic	30.1	10.6	10.6
Lard	0	76.4	7.5
EVOO	0	0	50
Walnuts	0	0	20
Fish oil	0	0	6
Cholesterol	0.03	0.98	0.21
Vitamin Mix, AIN-93-VX (94047)	10	10.6	10.6
Choline Bitartrate	2.5	2.65	2.65
Mineral Mix, AIN-93G-MX (94046)	35	37.15	37.15
Potassium Citrate, monohydrate	5	5.3	5.3
Magnesium Oxide	1.2	1.27	1.27
Ferric Citrate	0.3	0.32	0.32
Cupric Carbonate	0.01	0.011	0.011
**Fat profile**			
Saturated fat (g/kg)	8.71	35.8	20.6
Monounsatured fat (g/kg)	5.74	40.7	46.1
Polyunsaturated fat (g/kg)	26.1	19.6	29.0
Unsaturated to saturated fat ratio	3.65	1.68	3.65

Supplementary Table S2. Fatty acid profile of the virgin olive oil included in the MED. SFAs, saturated fatty acids; MUFAs, monounsaturated fatty acids; PUFAs, polyunsaturated fatty acids.

**Fatty acid**	**%**
C14:0	0,0
C16:0	14,4
c9-C16:1	1,2
C17:0	0,1
c9-C17:1	0,2

C18:0	2,0
c9-C18:1	70,2
c9,12-C18:2	10,5
C18:3 n3	0,6
C20:0	0,4
C20:1	0,3
C22:0	0,1
C24:0	0,1
**SFAs**	17,0
**MUFAs**	71,9
**PUFAs**	11,1
**TOTAL**	100

Supplementary Table S3. Nutritional information of the walnuts included in the MED. SFAs, saturated fatty acids; MUFAs, monounsaturated fatty acids; PUFAs, polyunsaturated fatty acids.

	100 g
Energetic value (Kcal)	685
Proteins (g)	15
Total fat (g)	65
SFAs (g)	6.4
MUFAs (g)	10
PUFAs (g)	48
Carbohydrates (g)	4.0
Sugars (g)	2.9
Dietary fiber (g)	12
Salt (g)	0.02

Supplementary Table S4. Nutritional information of MEG-3™ '30' n-3 Food Oil included in the MED.

Energy content total<br>Energy content total<br><br>**Content in:**	kJ/100 g<br>kcal/100 g	3'687<br>897
Fat	g/100 g	99.7
-> thereof saturated fatty acids	g	27.4
-> thereof mono-unsaturated fatty acids	g	22.5
-> thereof poly-unsaturated fatty acids	g	40.7
-> thereof trans fatty acids[1]	g	0
-> thereof cholesterol[1]	mg	530
Carbohydrates	g/100 g	0
-> thereof sugars	g	0
-> thereof polyols	g	0
-> thereof starch	g	0
Fibre	g/100 g	0
Protein	g/100 g	0

Salt (Sodium content x 2.5)	g/100 g	0

Supplementary Table S5. Identified brain N-glycans. Employed glycan nomenclature: F- Fucose; G- Galactose; S- N-Acetylneuraminic acid; Ga- a-linked Galactose; A1- Monoantennary, A2- Biantennary, B, bisecting GlcNAc linked α1-4 to α1-3 mannose. Numbers with parentheses indicate the preceding monosaccharide's linkage while those not in parentheses indicate the preceding characteristic's number. For example, F(6)A3G(4)3S(3,3,3)3 represents a core fucosylated triantennary glycan with 3 galactoses directly attached to antennae, and the three antennae terminated with an N-glycolylneuraminic acid. Employed glycan nomenclature for glycan composition: HexNAc- N-Acetylhexosamine; Hex- Hexose; NeuAc- N-Acetylneuraminic acid; Fuc- Fucose; Gal- Galactose; GlcNAc- N-Acetylglucosamine; Man- Mannose.

Name	Composition	Precursor m/z
-	HexNAc2 Hex3	611.7628
M4	GlcNAc2 Man4	692.7899
M5	GlcNAc2 Man5	773.8161
-	HexNAc2 Hex4 NeuAc1	816.7985
F(6)M5	Fuc1 GlcNAc2 Man5	846.8442
M6 D1 (putative)	GlcNAc2 Man6	854.8422
M6D3	GlcNAc2 Man6	854.8434
F(6)A2	Fuc1 GlcNAc4 Man3	887.8706
A2[6]G(4)1	Gal1 GlcNAc4 Man3	895.872
A3	GlcNAc5 Man3	916.3829
M7 (putative)	GlcNAc2 Man7	935.8692
M7D3	GlcNAc2 Man7	935.8699
F(6)A2[3]G(4)1	Gal1 Fuc1 GlcNAc4 Man3	968.8977
A2G(4)2	Gal2 GlcNAc4 Man3	976.8975
F(6)A3	Fuc1 GlcNAc5 Man3	989.411
-	HexNAc4 Hex5 Fuc1 NeuAc3	991.3839
-	HexNAc7 Hex7 Fuc1	1011.0655
-	HexNAc3 Hex4 Fuc1 NeuAc1	1012.9059
F(6)A3G(4)3S(3,3)2	NeuAc2 Gal3 Fuc1 GlcNAc5 Man3	1016.0619
M8 D2,D3	GlcNAc2 Man8	1016.8943
M8	GlcNAc2 Man8	1016.8956
F(6)M5A1G(4)1	Fuc1 Gal1 GlcNAc3 Man5	1029.4121
	HexNAc8 Hex7	1029.7350
-	HexNAc6 Hex7 Fuc1 NeuAc1	1040.0698
-	NeuAc1 Hex1 HexNAc4 Hex3	1041.9283
F(6)A2[3]G1Ga1	Fuc1 Gal2 GlcNAc4 Man3-Isomer 1	1049.9238
F(6)A2[6]G1Ga1	Fuc1 Gal2 GlcNAc4 Man3-Isomer 2	1049.9261
-	HexNAc5 Hex6 NeuAc3	1064.7501
F(6)A2[3]BG(4)1	Gal1 Fuc1 GlcNAc5 Man3	1070.4368
-	HexNAc6 Hex7 NeuAc2	1088.4155
F(6)A4	Fuc1 GlcNAc6 Man3	1090.9519
-	HexNAc3 Hex5 Fuc1 NeuAc1	1093.9325
M9	GlcNAc2 Man9	1097.9217

F(6)M4A1G(4)1Sg(6)1	Fuc1 Gal1 GlcNAc3 Man4 NeuGc1	1101.9299
-	HexNAc3 Hex6 NeuGc1	1110.419
F(6)A3G(4)3S(3,3,3)3	NeuAc3 Gal3 Fuc1 GlcNAc5 Man3	1113.0953
F(6)A2[6]G(4)1S(6)1	Fuc1 Gal1 GlcNAc4 Man3 NeuAc1-Isomer 1	1114.4457
F(6)A2[3]G(4)1S(3)1	Fuc1 Gal1 GlcNAc4 Man3 NeuAc1-Isomer 2	1114.4462
-	HexNAc6 Hex5 Fuc1 NeuAc3	1126.7695
-	HexNAc8 Hex8 Fuc1	1132.4403
-	HexNAc5 Hex4 NeuAc1	1143.4665
-	HexNAc5 Hex6 NeuAc4	1161.7798
-	HexNAc6 Hex6 Fuc1 NeuAc3	1180.7875
-	HexNAc4 Hex5 Fuc1 NeuAc1	1195.9816
-	HexNAc5 Hex6 Fuc1 NeuAc4	1210.1249
-	HexNAc5 Hex4 Fuc1 NeuAc1 -Isomer 1	1215.9866
F(6)A2[6]BG(4)1S(6)1	NeuAc1 Gal1 Fuc1 GlcNAc5 Man3-Isomer 2	1215.9891
F(6)A4G(4)4S(3,3,3)3	NeuAc3 Gal4 Fuc1 GlcNAc6 Man3-Isomer 1	1234.8057
F(6)A3G(4)3Lac1S3	NeuAc3 Gal4 Fuc1 GlcNAc6 Man3-Isomer 2	1235.1454
M11 a3D1,[D2(1),D3(1)],a2D4(2)	GlcNAc2 Man11	1259.9943
-	HexNAc4 Hex5 NeuAc2	1268.4994
-	HexNAc6 Hex7 NeuAc4 -Isomer 1	1283.491
-	HexNAc6 Hex7 NeuAc4 -Isomer 2	1283.4952
-	HexNAc8 Hex9 Fuc3	1283.8297
A2G(4)2Sg(3,6)2	NeuGc2 Gal2 GlcNAc4 Man3- Isomer 1	1283.9852
A2G(4)2Sg(6,6)2	NeuGc2 Gal2 GlcNAc4 Man3- Isomer 2	1283.9866
-	HexNAc7 Hex7 Fuc1 NeuAc3	1302.8351
A3G(4)3S(6)1	NeuAc1 Gal3 GlcNAc5 Man3	1304.9926
-	HexNAc6 Hex5 Fuc2	1325.9973
FA4G(4,4,4,4)4S(3,3,3,3)4	NeuAc4 Gal4 Fuc1 GlcNAc6 Man3	1331.838
F(6)A2G(4)2S(6,6)2	NeuAc2 Gal2 Fuc1 GlcNAc4 Man3-Isomer 1	1341.0211
F(6)A2G(4)2S(3,3)2	NeuAc2 Gal2 Fuc1 GlcNAc4 Man3-Isomer 2	1341.0214
F(6)A2G(4)2Sg(6,6)2	Fuc1 Gal2 GlcNAc4 Man3 NeuGc2-Isomer 1	1357.016
F(6)A2G(4)2Sg(3,6)2	Fuc1 Gal2 GlcNAc4 Man3 NeuGc2-Isomer 2	1357.5054
-	HexNAc5 Hex4 NeuAc2 Fuc1	1361.5341
-	HexNAc5 Hex5 NeuAc2	1370.0424
F(6)A3G(4)3S(3)1	Fuc1 Gal3 GlcNAc5 Man3 NeuAc1	1378.0222
-	HexNAc6 Hex7 Fuc2 NeuAc4	1380.5217
	HexNAc6 Hex3 Fuc1 NeuAc2	1382.0477
-	HexNAc4 Hex5 NeuAc3	1414.0498
F(6)A2BG(4)2S(6,6)2	NeuAc2 Gal2 Fuc1 GlcNAc5 Man3	1442.5621

-	HexNAc5 Hex6 Fuc1 NeuAc3	1669.6472
